# Application of Decision Tree in PE Teaching Analysis and Management under the Background of Big Data

**DOI:** 10.1155/2022/8091838

**Published:** 2022-07-08

**Authors:** Yong Che, Kaixuan Che, Qinlong Li

**Affiliations:** ^1^Department of Public Education, Anyang Preschool Teachers College, Anyang, Henan 456150, China; ^2^Exercise Science School, Beijing Sport University, Beijing 100084, China

## Abstract

With the continuous development of physical education reform, the defects and deficiencies of physical education teaching in colleges and universities are increasingly exposed. The reform of the original physical education teaching thought, education system, teaching mode, and method has achieved little. At present, the research on the prediction of physical education teaching achievements is mainly aimed at the prediction of athletes and physical education teaching achievements or the prediction of the past data of college students. This paper studies the physical education under the decision tree under the background of big data and constructs the physical education management system. When the number of tests reaches 40, the qualified rate of long-distance running is 65.2%, that of basketball is 68.1%, and that of volleyball is 68.2%. The quality of physical education teaching determines the lifeline of the development of school physical education teaching. In the process of collecting and selecting teaching materials, this paper enriches teaching materials and teaching reflection, cultivates one's own understanding, and improves the artistic appeal and creativity of teaching.

## 1. Introduction

As the grass-roots management of schools, the purpose of PE teaching management is to maximize the teaching efficiency. Improving the efficiency of PE teaching has become the starting point and destination of PE teaching management. Through modern means, the PE teaching management can be standardized, automated, and networked. The reform of the original PE teaching thought, education system, teaching mode, and methods has little effect [1, 2]. At present, in China's education, teachers are the center of high schools in most areas to strictly manage students, while students are the center after college, which requires students to have a high degree of consciousness [3]. This gap has led to a significant decline in some students' performance, and the performance prediction system has become the main means to prevent and solve this problem [4, 5]. However, due to the influence of many factors on university students' academic performance, the shortage of manpower in the prediction system, and the influence of human factors on the prediction results, the traditional comprehensive performance prediction strategy of university students has some problems such as low efficiency and poor prediction accuracy [6]. The teaching mode of PE teaching is very urgent and necessary and has a wide range of practical and guiding significance. The research on the teaching mode of PE teaching is essentially the research on the overall and reasonable procedure of the teaching process of PE teaching. Through the research on the teaching mode, it can basically reflect the core content of the reform of PE teaching [5, 7].

At present, the research on the prediction of PE teaching achievements is aimed at the prediction of athletes and PE teaching achievements or the prediction of college students' past data. It gives the specific implementation process to realize the prediction of PE teaching performance [8, 9]. The decision tree algorithm under big data determines the attribute with the best classification of the training set among the conditional attributes [10].

As an important technical means in big data, the decision tree algorithm integrates the technologies of machine learning, data statistics, intelligent database, neural network, and so forth. It mines the potential data of related influencing factors through the algorithm and provides an ideal model of modern teaching evaluation database for educational management and decision-making [11, 12]. By considering the factors that affect PE teaching, the decision tree is constructed, the comprehensive evaluation of the influencing indicators is realized, and targeted solutions to improve the instructional quality are given [13]. Applying the decision tree under big data to explore the modern PE teaching evaluation system, providing the ideal model hidden in the modern PE teaching evaluation database for PE teaching administrators and decision makers has certain theoretical significance and application value for promoting the information management of PE teaching and improving the quality of school PE teaching [14]. The decision tree technology in big data is introduced into the evaluation system of PE instructional quality, with the aim of promoting the theoretical research of PE instructional quality evaluation and improving the quality of PE teaching.

## 2. Related Work

Wang et al. [15] proposed that there is a lack of clear teaching objectives in PE teaching. Although, in the process of continuous promotion of instructional reform in recent years, education on physical fitness and moral quality has been added in PE teaching curriculum, there is no clear positioning in the setting of specific talent training objectives [15]. Silverman [17] put forward that PE teaching is a very complex professional business activity, involving many factors, such as teachers, students, managers, and teaching conditions [16]. Vercruysse et al. proposed that the application of computer and network integrated technology can reduce the labor intensity of PE teaching teachers and managers in school PE teaching management, improve work efficiency, and promote the reform and development of school PE teaching. Therefore, it is more and more urgent to apply sports technology to network teaching [17]. Ward et al. [18] proposed that the development of society is inseparable from the promotion of talents. As the cradle of cultivating talents, it is imperative to speed up the reform of PE teaching mode under the guidance of professional physical fitness, but there are some difficulties in this process. Therefore, it is necessary to constantly strengthen the research on PE teaching mode under the guidance of professional physical fitness [18]. Meendering et al. [19] proposed that the management of students in PE teaching is mainly carried out through teachers' PE teaching classes and related activities. This includes the management of students' organization, compliance with teaching conventions, attendance, physical condition, learning performance, coordination, and control in the teaching process [19]. Curran and Standage [20] put forward new ideas, as well as the overall instructional reform and experiment under its guidance, including PE teaching objectives, teaching contents, teaching methods, organizational forms, and teaching means, and gradually established one group or even several groups, in line with the spirit of reform and the teaching reality of colleges and universities in China's teaching procedures with strong operability and systematization, corresponding structure and function of PE teaching curriculum and teaching process structure, and framework or type [20]. Gu and He [21] put forward that there are still many problems in the current PE teaching in higher vocational colleges, and the teaching ideas cannot keep up with the development requirements of the times. When carrying out PE teaching, they are greatly influenced by the traditional teaching ideas, and the teaching methods are mainly classroom teaching, focusing on the teaching of knowledge points in textbooks, but there is a lack of teaching on physical skills [21]. Li et al. [22] put forward that the PE teaching process is a process of gradual improvement, and the management of this process should be continuous, uninterrupted, and disjointed, and some routine cannot be changed casually. From the time point of view, although it is divided into different links and stages, the teaching management cannot be relaxed, and it is necessary to persevere [22]. Wang et al. [23] put forward that expanding the enrollment scale of colleges and universities is an important strategy for the transformation of which has a very positive significance. However, it is undeniable that it also has negative aspects that affect the quality of higher education. Under this environment, the decline of instructional quality in college PE teaching is more serious [23]. Fang L et al. put forward that the teaching content of PE teaching is unscientific, and teaching is to cultivate applied talents. Therefore, the teaching content of the course must be targeted in terms of occupation. However, it is obvious that this point is ignored in the current PE teaching process, and the setting of teaching content is unscientific, which has a great impact on the cultivation of students' sports consciousness [24]. At present, many schools pay close attention to management, and each school has strict requirements on its teaching methods, classroom discipline, and teaching arrangements and even constantly updates them. The campus system is filled in to strengthen management and it is hoped to achieve the education standard with the help of the seriousness and meticulous management. However, in such strict management, the teaching effect of some schools is sometimes criticized because it cannot achieve the desired effect or even backfire. It can be seen that, under the strict teaching management, there are still many problems in principle which we need to find out.

This paper studies the analysis and management of PE teaching under the background of big data. Because of its asynchronous teaching characteristics of rich teaching materials and unlimited learning time, PE teaching can undertake learning outside the PE teaching classroom and there cab be an extension of the PE teaching classroom to expand students' learning content and learning time. Teachers can also guide students' after-school physical exercise and learning. In this paper, the construction of sports information management system is based on the security of the network, which is fully considered by the operators. The PE teaching management system under the decision tree mainly includes teaching guiding ideology, teaching objectives, teaching structure, teaching system, teaching content, syllabus, teaching organization form, teaching methods, teaching means, teaching evaluation, and related conditions.

## 3. Research on Decision Tree Algorithm under the Background of Big Data

### 3.1. Algorithm and Model of Decision Tree

Because of the huge dataset, it is impossible to process all the data in one time when calculating in memory, and a lot of data needs to be temporarily stored in disk. Because the decision tree algorithm needs to read and write data, the huge data makes the reading and writing speed slow. The training process of decision tree is iterative splitting and growing from the root node to the bottom, layer by layer. Regulating the construction of the whole tree is mainly used to train the tree model of big data. The controller can be involved in the computing cluster, and it is an independent computer, which performs distributed computing through MapReduce. When designing the development system architecture, on the one hand, we should combine the user's needs and select the appropriate architecture according to the requirements definition; on the other hand, when selecting the system architecture, we should consider the maturity and foresight of the current architecture.

The main reason why decision tree algorithm is so popular in data analysis and data mining applications is that the construction of decision tree does not need any domain knowledge, which is very suitable for exploratory knowledge mining, and it can process high-dimensional data. Among numerous data mining and statistical analysis algorithms, the greatest advantage of decision tree is that it produces a series of rules from root to branch (or leaf), which can be easily understood by analysts and business personnel. Moreover, these typical rules even need to be sorted out slightly, which are ready-made business optimization strategies and business optimization paths that can be applied. In addition, decision tree technology is very tolerant to the distribution or even lack of data and is not easily affected by extreme values.

**Figure 1:**
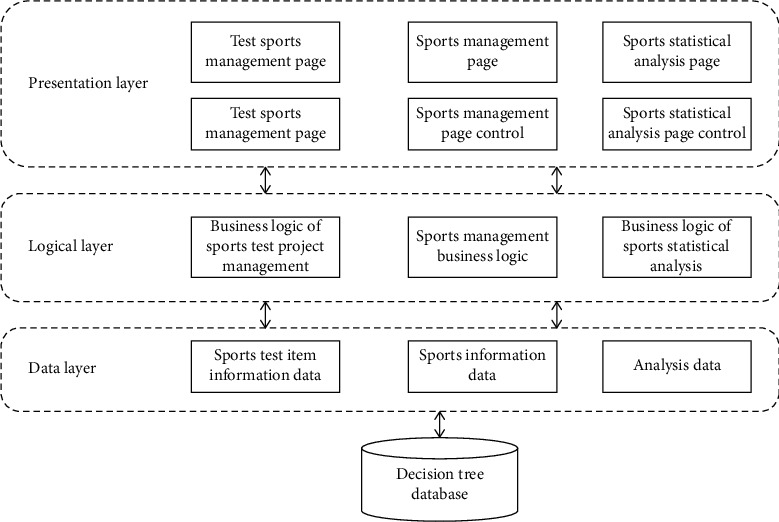
Structure diagram of PE teaching management system based on decision tree algorithm.

The architecture of PE teaching management system based on decision tree algorithm is shown in Figure 1.

Using the basic idea of decision tree algorithm, the attribute with the largest information gain rate is selected as the splitting attribute to form branches, and then the algorithm is called recursively for each branch until it cannot split a new branch. The calculation formula of information gain rate is as follows:

(1) $$\begin{array}{c} \text{GainRatio}\left(A\right)=\frac{\text{Gain}\left(A\right)}{\text{Split}I\left(A\right)}. \end{array}$$

In the above formula, the information gain Gain(*A*) represents the information gain of attribute *A*, which can be calculated according to the information gain in the decision tree algorithm. Split*I*(*A*) is called split information, and its calculation method is as follows:

(2) $$\begin{array}{c} \text{Split}I_{A}\left(D\right)=-\sum_{j=1}^{v}\frac{\left|e_{r}\right|}{D}\times \log_{2}\left(\frac{\left|e_{r}\right|}{D}\right). \end{array}$$

The training dataset *D* has *m* classes *C*_*i*_ (*i*=1,2,…*m*). If *A* is a split attribute and has *v* values *v*_1_, *v*_2_,…*v*_*v*_, *D* is divided into *v* subsets {*e*_1_, *e*_2_,…, *e*_*v*_}, and the number of tuples belonging to class *C*_*i*_ in subset *e*_*r*_ is |*e*_*r*_|; then the probability *p*_*i*_ belonging to class *C*_*i*_ is |*e*_*r*_|/|*D*|.

For a training sample, the error between the actual output and the expected output can be defined as

(3) $$\begin{array}{c} \text{Error}=\frac{1}{2}\sum_{k=1}^{c}{\left(T_{k}-O_{k}\right)}^{2}, \end{array}$$

where *c* is the number of output layer units, *Tk* is the expected output of output layer unit *k*, and *O*_*k*_ is the actual output of output layer unit *k*.

Let *S* be a set of *s* data samples, the class attribute has *m* different classes *C*_*i*_, and *s*_*i*_ is the number of samples contained in *C*_*i*_ class, and the information entropy required to classify a given sample is given by the following formula:

(4) $$\begin{array}{c} I\left(s_{1},s_{2},\ldots ,s_{m}\right)=-\sum_{i=1}^{m}p_{i}\log_{2}\left(p_{i}\right), \end{array}$$

where *p*_*i*_ is the probability that any sample belongs to *C*_*i*_, and it is estimated by *s*_*i*_/*s*.

**Figure 2:**
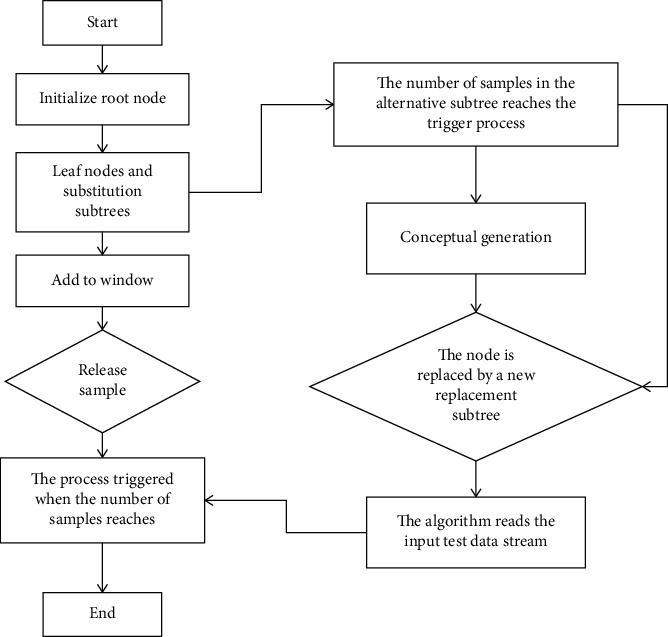
Flow chart of decision tree algorithm.

The law of data is changing over time. New data may not match the old parameters well, and the model is also changing over time. A single model is difficult to fully describe the data. Therefore, how to update the decision-making model according to the trend of data change is also the development direction of decision tree algorithm model in the future. In order to solve the problems of limited memory and training efficiency faced by the streaming data classification algorithm, the most effective way is to use the divide-and-conquer method to decompose the original computing task into several identical subtasks to deal with, so that each computer node can balance the load. Through the established decision tree model, each instance in the test data stream is predicted in real time, and the prediction results are matched with the class labels printed in advance, and then the matching results are inserted into the adds window to detect the concept drift. The detailed growth process of the decision tree algorithm is shown in Figure 2.

Let the dataset *S* contain *s* data samples, and let the category attributes take *m* values, corresponding to *m* different categories *c*_*i*_,  *i*=1,2,…, *m*. If *s*_*i*_ is set as the number of samples in category *c*_*i*_, then the amount of information required to classify a given sample is

(5) $$\begin{array}{c} I\left(s_{1},s_{2},\ldots ,s_{m}\right)=\sum_{i=1}^{m}-p_{i}\log_{2}\left(p_{i}\right), \end{array}$$

where *p*_*i*_=*s*_*i*_/*s* is the probability that any sample belongs to category *c*_*i*_.

If *A* is selected as the test attribute and there are *v* values {*a*_1_, *a*_2_,…*a*_*v*_}, *S* is divided into *v* subsets {*S*_1_, *S*_2_,…, *S*_*v*_}, where *S*_*j*_ contains the data samples whose attribute *A* in set *S* takes *a*_*j*_*L* value. Assuming that *S*_*ij*_ is the number of samples belonging to category *c*_*i*_ in subset *S*_*j*_, the information entropy required to divide the current sample set by attribute *A* is used.

(6) $$\begin{array}{c} E\left(A\right)=\sum_{j=1}^{v}\frac{\left(s_{1j}+s_{2j}+\cdots +s_{mj}\right)}{s}\times I\left(s_{1j},s_{2j},\ldots ,s_{mj}\right)=-\sum_{j=1}^{v}\sum_{i=1}^{m}\frac{s_{1j}+s_{2j}+\cdots +s_{mj}}{s}. \end{array}$$

*A* attributes the information gain obtained by the set partition of the current branch node.

(7) $$\begin{array}{c} \text{gain}\left(A\right)=I\left(s_{1},s_{2},\ldots ,s_{m}\right)-E\left(A\right). \end{array}$$

According to the knowledge of limit theory, when 0 ≤ *x* ≤ 1, *n* tends to infinity, and, with the increase of the power degree in the above formula, the result after the third term will become smaller and smaller. Compared with the first two terms, the latter terms are approximately 0, and the final function *f*(*x*) is simplified to

(8) $$\begin{array}{c} \text{In}\left(1+x\right)=x-\frac{x^{2}}{2}. \end{array}$$

From the definition in the previous decision tree algorithm, the calculation formula of information amount is

(9) $$\begin{array}{c} I\left(s_{1},s_{2},\ldots ,s_{n}\right)=-\sum_{i=1}^{n}p_{i}\log_{2}p_{i}=-\sum_{i=1}^{n}\frac{s_{i}}{s}\log_{2}\frac{s_{i}}{s}. \end{array}$$

The above formula is further simplified as

(10) $$\begin{array}{c} I\left(s_{1},s_{2},\ldots ,s_{n}\right)=-\sum_{i=1}^{n}\frac{s_{i}}{s}\log_{2}\left(1+\left(-\frac{s-s_{i}}{s}\right)\right). \end{array}$$

Through the input training sample, the method of inserting decision leaf node is called to insert the training sample from the root node to the corresponding leaf node, and the statistical information of the leaf node is updated. If all the arrived samples belong to the same class, the method of splitting the leaf node is called, in which the number of samples arrived by the leaf node is artificially set to avoid continuous calculation. If the calculation results meet the constraints and other corresponding requirements, split is executed, and the number of newly added leaf nodes is the number of the decision attributes of the leaf nodes.

### 3.2. Realization of PE Teaching Management System

At present, most public PE teaching courses in universities in China adopt the option teaching method. Although this teaching mode has very positive significance compared with the previous unified teaching, there are still some problems in the setting of teaching content, the arrangement of teaching hours, and the guidance of after-school PE teaching. This paper constructs a PE teaching management system under the BIG DATA background decision tree. In terms of teaching guiding ideology, it pays attention to imparting basic sports knowledge, basic technology, and skills, as well as enhancing students' physique, and the teaching organization form is mainly the original teaching class. Such curriculum can relatively meet students' interest needs and meet the development trend of modern education, but there are still some problems that are difficult to solve in practical operation, such as the division of classes at the beginning of the semester and the submission of students' scores by departments at the end of the semester. From the perspective of structure in the teaching management system, the data types include semistructured, unstructured, and structured. In order to unify and integrate multichannel data and facilitate later BIG DATA processing, it is necessary to normalize it and preprocess it and finally store it in the BIG DATA teaching management system. The pretreatment, management, and collection of teaching status data in colleges and universities need to compare the connotation of data. Check the data sources one by one and provide data support materials for important data. At present, the teaching status data acquisition system in both undergraduate colleges and higher vocational colleges uses the stand-alone version, which has the advantages of good data confidentiality and simple operation.

## 4. Experimental Results and Analysis

25 students and 5 PE teaching teachers were randomly selected to participate in the system performance evaluation test. The PE teaching and learning information of 25 students was completely entered in the system. Students and teachers use three kinds of systems for routine operation and generate intelligent teaching decision-making scheme. In the test, the response time required for users to log in to the main function interface of the system is recorded. After the test, the satisfaction generated by students and teachers using the system and the evaluation of the rationality of intelligent teaching decision-making are understood in the form of questionnaire. Through the first step of operation, the students' PE teaching scores are expressed by “pass” or “fail.” On this basis, the numbers of passing students and failing students in each sports test item are counted. The results are shown in Tables 1 and 2.

As shown in Tables 1 and 2, the average response time of the system entering the main function interface is 0.31 s, indicating that the system has no obvious stuck problem, and the response speed is faster than those of the other two systems, and the sensitivity of the system entering the main function interface is higher. Students' and teachers' satisfaction levels with the system reached 96.5% and 95.7%, respectively, which were excellent. However, the distributed intelligent management system of PE teaching in colleges and universities achieved only 87.8% satisfaction from teachers, which showed that the system interface design layout, decision-making accuracy, function setting, and other aspects did not meet the requirements of teachers.

**Figure 3:**
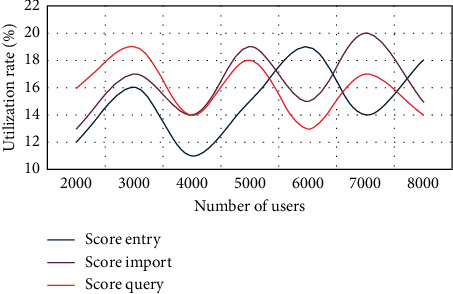
Changes of test data, utilization, and number of users.

In order to accurately record the changes of utilization and the number of users, score entry, score import, and score query are carried out to record the changes of indicators in each stage. The test records the operation process of performance management. The changes of test data, utilization, and the number of users are shown in Figure 3.

As can be seen from Figure 3, when the number of users is 5000, the utilization rate of score entry is 15.5%, the utilization rate of score import is 19.3%, and the utilization rate of score query is 18%. Only with the increase of the number of users is the utilization rate reached at the time of users. Nevertheless, the utilization rate is within the allowable range.

**Figure 4:**
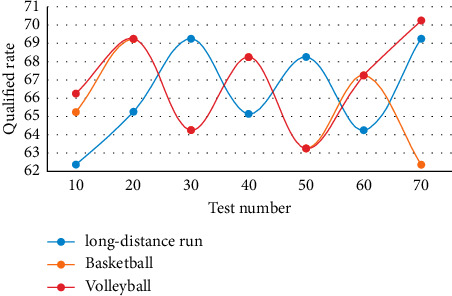
Qualification rate of different sports.

In this experiment, students' scores of Class 1, Class 2, and Class 3 were selected as training sets by the method of overall sampling. Long-distance running, basketball, and volleyball were tested, with a total of 150 records. The scores of these three classes were copied to the training example worksheet. Using the function, the number of people who passed and failed in a single subject in the training set was found out. The experimental results are shown in Figure 4.

As can be seen from Figure 4, when the test number reaches 40, the qualified rate of long-distance running is 65.2%, that of basketball is 68.1%, and that of volleyball is 68.2%. The quality of PE teaching determines the lifeline of school PE teaching development. To have a team of high-quality PE teaching teachers, we must have a complete evaluation system of PE instructional quality.

In order to determine the research direction and feasibility of this topic, Delphi method was used to issue three rounds of consultation questionnaires to experts in education and sports. According to the survey statistics, “very important,” “important,” “relatively important,” “average,” and “irrelevant” were assigned to 5, 4, 3, 2, and 1, respectively. According to the evaluation results of the first round of expert consultation questionnaire, the indicators with lower average value were excluded item by item and sorted out, a questionnaire was made and returned to each expert to evaluate the recovery of the expert consultation questionnaire again, as shown in Table 3. Experts evaluated the structural validity and content validity, as shown in Tables 4 and 5.

From the results of expert consultation and evaluation, the structural validity is 85.74% and the content validity is 88.33%, indicating that this questionnaire has high structural validity and content validity and is considered a formal questionnaire. The satisfaction of college PE teaching intelligent management system based on data warehouse is less than 85%, which is also not ideal.

**Figure 5:**
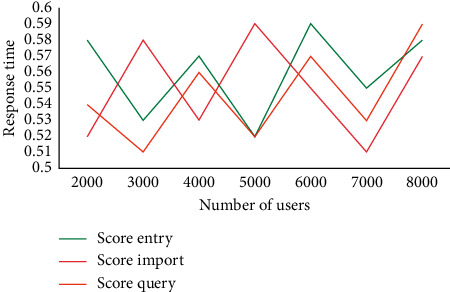
Change of test data, average response time, and number of users.

In order to accurately record the relationship between each performance index and the number of people, the changes of indexes in each stage are recorded by grade entry, grade import, and grade inquiry. The test records the operation process of performance management, and the changes of test data, average response time, and number of users are shown in Figure 5.

**Figure 6:**
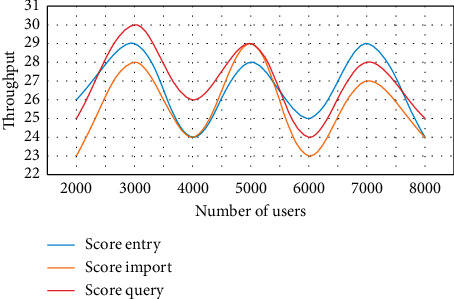
Changes in test data, throughput, and number of users.

As can be seen from Figure 5, when the number of users increases, the response time of score query suddenly increases to about 2.7 seconds, but it does not exceed 6 seconds. This shows that even if the maximum number of users is reached, the changes of throughput and number of users, score entry, score import, and score query are carried out, respectively, to record the changes of indicators in each stage. The test records the operation process of performance management. The changes of test data, throughput, and number of users are shown in Figure 6.

As can be seen from Figure 6, when there are 4,000 users, the throughput of score entry is 24, the throughput of score import is 24, and the throughput of score query is 26. When there are 6,000 users, the throughput of score entry is 254, the throughput of score import is 23, and the throughput of score query is 24. This shows that the business processing ability meets the requirements under the premise of the maximum number of users.

**Figure 7:**
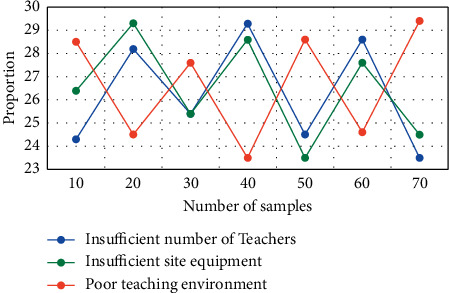
Changes of different factors in PE teaching management.

At present, physical education teaching generally only pays attention to the management of physical education teaching methods but lacks a clear evaluation system for the corresponding management effect and management system. This problem exists in the majority of colleges and universities. At this stage, the performance of this issue is also multifaceted. In the process of making corresponding teaching plans, the relevant departments of the school lack the cognition of corresponding teaching results and even neglect monitoring them. This experiment aims at the main problems existing in the management of PE teaching in colleges and universities, such as the shortage of teachers, the shortage of venues and equipment, and the bad teaching environment. The experimental results are shown in Figure 7.

Figure 7 shows the changes of three different factors in the teaching environment. Among the main problems existing in the management of physical education in colleges and universities, the number of teachers, the content changes of on-site equipment, and teaching environment are the main factors. It can be seen from Figure 7 that, among the main problems existing in the management of PE teaching in universities, when the number of test samples is 40, the proportion of insufficient teachers is 29.3%, the proportion of insufficient venues and equipment is 28.8%, and the proportion of poor teaching environment is 23.5%. The proportions of the three factors are almost the same. In the process of collecting and screening materials, enriching teaching materials, and teaching reflection, their own understanding is cultivated and the artistic appeal of teaching expressiveness and creativity is improved.

## 5. Conclusions

The way of physical education teaching will change from “teacher centered” to “student autonomy,” reflecting the characteristics of subjectivity and modernity. This paper studies the physical education under the decision tree under the background of big data and constructs the physical education management system. When the number of tests reaches 40, the qualified rate of long-distance running is 65.2%, that of basketball is 68.1%, and that of volleyball is 68.2%. In the process of realizing informatization, colleges and universities should learn from each other. Guided by the spirit of the report of the 17th National Congress of the Communist Party of China, we should thoroughly implement the “Scientific Outlook on Development” and promote the reform and development of physical education teaching in colleges and universities in Henan Province. The decision tree algorithm under big data should highlight the diversity and acceptability of physical education teaching content. From the perspective of physical education curriculum, the text curriculum should be transformed into students' own curriculum. In this paper, the decision tree technology in big data (hereinafter referred to as big data) is introduced into the PE teaching quality evaluation system, which promotes the theoretical research of PE teaching quality evaluation and improves the PE teaching quality. However, there are still some limitations in the research. The research has not evaluated the individual abilities of different athletes, so further analysis is needed in the future research.

## Figures and Tables

**Table 1:** Training set data.

	Long-distance run	Basketball	Short-distance run	Volleyball	Shot	High jump
Number of passing students	54	65	100	121	106	98
Number of failing students	103	92	58	38	53	62

**Table 2:** Performance evaluation of PE teaching management system.

Algorithm	System response time	Student satisfaction (%)	Teacher satisfaction (%)	Management rationality (%)
System	0.31	96.5	95.7	97.7
Distributed intelligent management system of PE teaching in colleges and universities	0.83	90.3	87.8	93.1
Intelligent management system of college PE teaching based on data warehouse	0.58	88.3	88.3	90.3

**Table 3:** Statistical table of recovery of expert consultation questionnaire.

Round	Number of copies issued	Number of recovered copies	Rate of recovery (%)
1	22	17	84
2	16	15	91.25
3	15	15	98

**Table 4:** Evaluation form of structural validity of expert consultation questionnaire.

Evaluation score	5	4.85	4	3.5	3	2
Corresponding number	3	6	4	3	1	1
Total number	15	Average score: 4.56	Construct validity: 85.75%			

**Table 5:** Evaluation form of content validity of expert consultation questionnaire.

Evaluation score	5	4.85	4	3.5	3	2
Corresponding number	4	6	3	3	0	1
Total number	15	Average score: 4.36	Construct validity: 88.35%			

## Data Availability

The data used to support the findings of this study are included within the article.

## References

[1] Mercier K., Centeio E., Garn A., Erwin H., Marttinen R., Foley J. (2021). Physical education teachers’ experiences with remote instruction during the initial phase of the COVID-19 pandemic. *Journal of Teaching in Physical Education*.

[2] Kaschalk-Woods E., Fly A. D., Foland E. B., Dickinson S. L., Chen X. (2021). Nutrition curriculum training and implementation improves teachers’ self-efficacy, knowledge, and outcome expectations. *Journal of Nutrition Education and Behavior*.

[3] Yang S. (2021). Construction of video courses of physical education and health education in colleges and universities under the MOOC platform. *Mobile Information Systems*.

[4] Morley D., Banks T., Haslingden C. (2021). Including pupils with special educational needs and/or disabilities in mainstream secondary physical education: a revisit study. *European Physical Education Review*.

[5] Nankwanga A., Annet K., Ismail A. (2021). Journal of PE teaching and Sport Management Assessment of the practices and perceptions towards age estimation among the sports fraternity in Uganda. *Journal of Athletic Training*.

[6] Harris N., Warbrick I., Atkins D. (2021). Feasibility and provisional efficacy of embedding high-intensity interval training into PE teaching lessons: a pilot cluster-randomized controlled trial. *Pediatric Exercise Science*.

[7] Hastie P. A., Wang W., Liu H., He Y. (2021). The effects of play practice instruction on the badminton content knowledge of a cohort of Chinese physical education majors. *Journal of Teaching in Physical Education*.

[8] Pike Lacy A. M., Eason C. M., Stearns R. L., Casa D. J. (2021). Secondary school administrators’ knowledge and perceptions of the athletic training profession, Part II: specific considerations for principals. *Journal of Athletic Training*.

[9] Corbett R. O., Harris P. C., Vela L., Saliba S. A., Hertel J. (2021). Athletic trainers’ perceptions of treating and managing patients with ankle sprains. *Journal of Athletic Training*.

[10] Pérez-Chiqués E., Strach P., Zuber K. (2021). Competing emergencies: a policy analysis of the opioid epidemic during COVID-19. *Journal of Comparative Policy Analysis: Research and Practice*.

[11] Lander N., Eather N., Morgan P. J., Salmon J., Barnett L. M. (2017). Characteristics of teacher training in school-based physical education interventions to improve fundamental movement skills and/or physical activity: a systematic review. *Sports Medicine*.

[12] Sun H., Li W., Shen B. (2017). Learning in physical education: a self-determination theory perspective. *Journal of Teaching in Physical Education*.

[13] Stankovic J. A., Sturges J. W., Eisenberg J. (2017). A 21st century cyber-physical systems education. *Computer*.

[14] Mooses K., Pihu M., Riso E. M., Hannus A., Kaasik P., Kull M. (2017). Physical education increases daily moderate to vigorous physical activity and reduces sedentary time. *Journal of School Health*.

[15] Wang H., Shen B., Bo J. (2021). Examining situational interest in physical education: a new inventory. *Journal of Teaching in Physical Education*.

[16] Silverman S. (2017). Attitude research in physical education: a review. *Journal of Teaching in Physical Education*.

[17] Vercruysse S. (2017). EFFECTIVELY TRAINING PE teaching TEACHERS TO IMPLEMENT INJURY PREVENTIVE STRATEGIES INTO THEIR PE LESSONS. *British Journal of Sports Medicine*.

[18] Ward P., Dervent F., Lee Y. S., Ko B., Kim I., Tao W. (2017). Using content maps to measure content development in physical education: validation and application. *Journal of Teaching in Physical Education*.

[19] Meendering J. R., Skinner M. M., McCormack L. A. (2021). Model school‐district wellness policies warrant improvements in comprehensiveness and strength. *Journal of School Health*.

[20] Curran T., Standage M. (2017). Psychological needs and the quality of student engagement in physical education: teachers as key facilitators. *Journal of Teaching in Physical Education*.

[21] Gu Z., He C. (2021). Application of fuzzy decision tree algorithm based on mobile computing in sports fitness member management. *Wireless Communications and Mobile Computing*.

[22] Li J., Lei H., Tsai S. B. (2021). Online data migration model and ID3 algorithm in sports competition action BIG DATA application. *Wireless Communications and Mobile Computing*.

[23] Wang F., Wang Q., Nie F., Yu W., Wang R. (2018). Efficient tree classifiers for large scale datasets. *Neurocomputing*.

[24] Fang L., Wei Q., Xu C. J. (2021). Technical and tactical command decision algorithm of football matches based on BIG DATA and neural network. *Scientific Programming*.

